# Results of Integrated Transmission Assessment Surveys for Lymphatic Filariasis and Malaria in Haiti, 2017–2022

**DOI:** 10.4269/ajtmh.23-0765

**Published:** 2024-06-25

**Authors:** Luccène Désir, Karen E. S. Hamre, Valéry Madsen Beau De Rochars, Jean F. Lemoine, Marc-Aurèle Telfort, Gregory S. Noland

**Affiliations:** ^1^ The Carter Center, Atlanta, Georgia;; ^2^ The Emerging Pathogens Institute and Department of Health Services Research, Management and Policy, University of Florida, Gainesville, Florida;; ^3^ Ministère de la Santé Publique et de la Population, Port-au-Prince, Haiti

## Abstract

Haiti is endemic for lymphatic filariasis (LF) and malaria, two mosquito-transmitted parasitic diseases targeted for elimination. The World Health Organization recommends a transmission assessment survey (TAS-1) to determine if LF prevalence is significantly beneath putative transmission thresholds (<2% antigen prevalence in Haiti, where *Culex* is the primary vector for *Wuchereria bancrofti*) to stop mass drug administration (MDA). Repeated TASs (TAS-2 and TAS-3) are recommended at 2–3-year intervals during post-treatment surveillance. From 2017 to 2022, The Carter Center assisted the Haitian Ministry of Public Health and Population in conducting 15 TASs in 11 evaluation units (EUs) encompassing 54 of the country’s 146 districts. Children 6–7 years old were assessed for circulating filarial antigen (CFA) by Filariasis Test Strip: *n* = 5,239 in TAS-1; *n* = 11,866 in TAS-2; and *n* = 1,842 in TAS-3, of whom eight (0.15%), 20 (0.17%), and eight (0.43%) tested positive, respectively. The number of positive results in children was less than the threshold in each EU. When available, participants (*n* = 16,663) were also tested for malaria by rapid diagnostic test, with 31 (0.19%) children testing positive for *Plasmodium falciparum*. Integrated TASs provided an efficient means to collect epidemiological data for LF and malaria in Haiti. Results indicated thresholds for stopping and maintaining the halt of MDA for LF have been achieved in all EUs, with the halt of MDA for 571,358 people in four districts and the first TAS-3 surveys conducted in Haiti. Investigations are needed to assess the potential of ongoing LF transmission, especially in areas where CFA-positive samples were detected in TAS-3.

## INTRODUCTION

The island of Hispaniola, shared by Haiti and the Dominican Republic, is the only remaining malaria-endemic island in the Caribbean, although Haiti accounts for nearly the entire population remaining in need of treatment of lymphatic filariasis (LF) in the Americas region.^1^^,^^2^ In 2006, the International Task Force for Disease Eradication (ITFDE) concluded that elimination of malaria and LF from Hispaniola was “technically feasible, medically desirable, and would be economically beneficial” to both countries.^3^ Since 2008, The Carter Center has provided technical assistance to the ministries of health in both countries to eliminate these two diseases.

Lymphatic filariasis is a vector-borne parasitic disease caused by infection with *Wuchereria bancrofti, Brugia malayi*, or *Brugia timori* and transmitted by multiple genera of mosquitoes. Lymphatic filariasis causes permanent and long-term disability because of its debilitating manifestations, which can include lymphedema, elephantiasis, genital diseases (hydrocele, chylocele), and acute febrile episodes of adenolymphangitis.^4^ These conditions are the result of chronic dysfunction caused by adult worms that inhabit the lymphatic vessels. Adult worms produce millions of infective microfilariae (MF) that appear in the circulatory system, predominantly at night. Treatment with albendazole (donated by GSK) in combination with either diethylcarbamazine citrate (DEC; donated by Eisai) or ivermectin (donated by Merck & Co., Inc.) impairs production of MF, thereby limiting transmission to mosquitoes.^5^^,^^6^ However, treatment does not kill the adult worms, meaning that multiple years of mass drug administration (MDA) with effective coverage (≥65%) of the total population is recommended to interrupt LF transmission.^7^ Recent studies have demonstrated the superiority of the triple-drug ivermectin-DEC-albendazole combination in achieving sustained MF clearance.^8^^,^^9^

The ITFDE considered LF as one of six potentially eradicable diseases in 1993,^10^ and in 1997 the World Health Assembly passed Resolution 50.29 calling for the elimination of LF as a public health problem globally.^11^ The World Health Organization (WHO) launched the Global Program to Eliminate LF in 2000 with two main goals: interrupt transmission through MDA and reduce the suffering of people living with LF through morbidity management and disability prevention to care for those already suffering from LF. At that time, nearly 1.1 billion people in 80 countries were at risk of infection, including seven countries in the Americas.^4^ Owing to the tremendous efforts of national programs, more than 9 billion treatments for LF have been delivered worldwide in 70 countries since 2000.^12^ Twenty-nine countries or territories have reduced infection prevalence to levels at which transmission is assumed not to be sustainable. Nineteen of these countries or territories have now been acknowledged as achieving the elimination of LF as a public health problem. In the Americas region, three countries (Costa Rica, Trinidad and Tobago, and Suriname) of the seven originally identified as endemic have been reclassified as nonendemic.^12^ The region continues showing progress, with Brazil^13^ and the Dominican Republic^14^ currently in post-treatment surveillance (PTS).

In Haiti, LF is caused by *Wuchereria bancrofti* and is transmitted by *Culex quinquefasciatus*, the only vector species in this region. Lymphatic filariasis is known to have been endemic since at least the mid-1700 s, likely the result of importation of enslaved Africans to the island of Hispaniola.^13^ The Haitian Ministry of Public Health and Population (MSPP) launched nationwide LF mapping surveys in 2001 using an adaptation of the lot quality assurance sampling (LQAS) method to detect circulating filarial antigen (CFA) by rapid immunochromatographic test. The overall population-weighted CFA prevalence among children 6–11 years old was 7.3%.^15^ Infected children were found in 117 (88.0%) of 133 communes (districts) at the time of the mapping, with infections more prevalent in the northern part of the country. Based on these results and subsequent investigations,^16^ the MSPP decided to implement annual MDA with DEC-albendazole to all communes in the country (currently numbering 146, though the national LF elimination program continues to consider the demarcations of 140 communes). Each commune is considered an implementation unit (IU). Mass drug administration started in 2000 in Leogane commune, and full geographic coverage of all communes was achieved in 2012.^17^^,^^18^

To determine whether LF transmission has been interrupted and MDA can safely stop, the WHO recommends a transmission assessment survey (TAS-1).^7^ A transmission assessment survey is an LQAS-type survey of children 6–7 years old residing in an evaluation unit (EU), which is composed of an IU or a group of epidemiologically similar IUs. An EU “passes” a TAS if the number of CFA-positive individuals is less than a cutoff value corresponding to putative transmission breakpoints (significantly <2% antigen prevalence in areas where *W. bancrofti* is transmitted by *Culex* or *Anopheles* mosquitoes). The WHO also recommends repeated surveys (TAS-2 and TAS-3) every 2–3 years for the purpose of PTS to monitor for recrudescence or reintroduction in areas that have stopped MDA. The MSPP conducted the first TAS-1 in 2012 in La Tortue Island. Since then, the MSPP has conducted numerous TASs with assistance from multiple implementing partners.

Malaria is a preventable and treatable disease.^19^ Despite this, in 2022 there were an estimated 249 million cases resulting in 608,000 deaths globally.^1^ Haiti is committed to eliminating malaria,^20^^,^^21^ and in 2009, it joined with the Dominican Republic to develop a binational plan for island-wide malaria elimination.^22^ Haiti’s malaria elimination strategies are based on the WHO’s core guidelines for prevention (including vector control and bed net distribution), case management (early diagnosis and prompt and effective treatment), and surveillance.^23^ A country may apply to the WHO to be certified as malaria-free after 3 consecutive years with zero autochthonous cases in the presence of a strong surveillance system that can prevent reestablishment of indigenous transmission.^24^

In 2022, Haiti reported 14,757 confirmed cases of malaria, which accounted for more than 97% of cases on Hispaniola.^1^ The primary vector of transmission is *Anopheles albimanus*, a relatively inefficient vector with both zoophilic and exophilic tendencies.^25^ Nearly all infections are caused by *Plasmodium falciparum*, which remains sensitive to chloroquine on the island. Transmission is perennial, with bimodal peaks generally occurring after the rainy seasons from November to January and from May to July. Overall malaria prevalence in Haiti consistently measures <1%.^26^^–^^29^ However, transmission is heterogeneous and focal, with reported estimates as high as 16.1% in North department and 40.7% and 46.3% in Grande Anse department,^30^^,^^31^ as detected by highly sensitive quantitative reverse-transcription polymerase chain reaction (PCR) testing among a sample of women seeking obstetric and gynecological care. These hotspots of transmission and a high proportion of asymptomatic carriers pose a challenge to transmission elimination. Following past MSPP surveys that integrated malaria testing in the context of LF TASs,^32^ this report describes results from integrated malaria–LF TASs conducted with assistance from The Carter Center from 2017 to 2022.

## MATERIALS AND METHODS

### Survey area and survey design.

**Figure 1.**
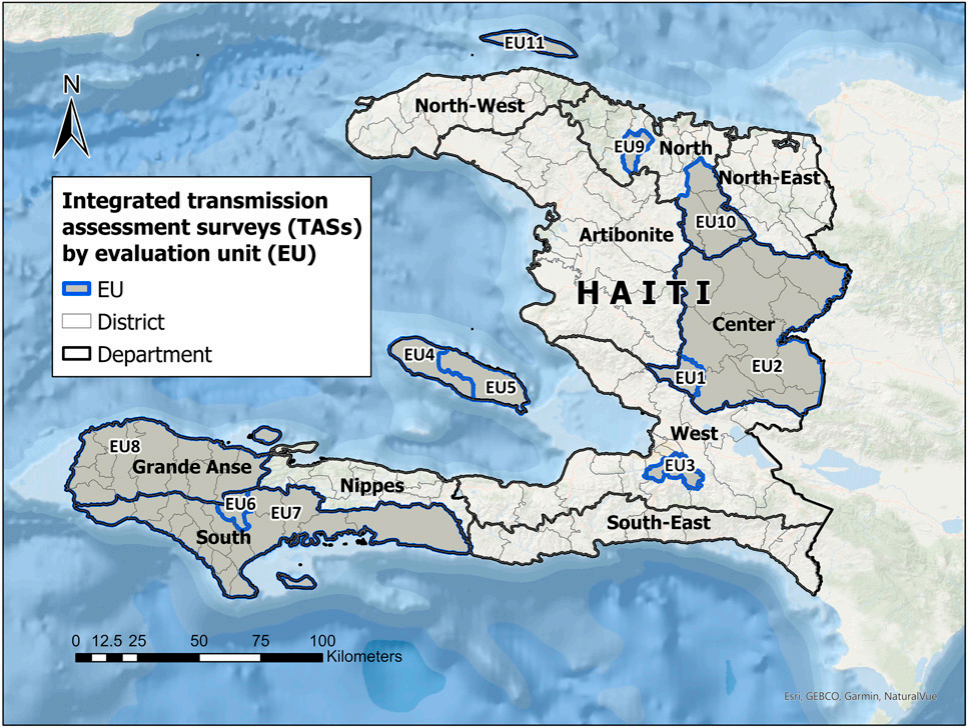
Geographic distribution of 11 evaluation units for integrated transmission assessment surveys in Haiti, 2017–2022.

Cross-sectional TASs were conducted in 54 communes across 6 of 10 regional departments in Haiti encompassing approximately 3,030,117 people (25% of the projected national total). Each commune successfully passed a pre-TAS by demonstrating CFA prevalence of <2% in one sentinel site and one spot-check site per commune. Communes were organized into 11 EUs, arbitrarily numbered EU1–EU11 here, based on geography and epidemiological similarity (Figure 1). Baseline prevalence and MDA history for each EU is shown in Table 1. The Carter Center supported a total of 15 TASs: TAS-1 in four EUs; TAS-2 in nine EUs; and TAS-3 in two EUs. Some EUs were included over multiple TASs, whereas other partners may have supported preceding TASs in areas assessed in this report.

**Table 1:** Inventory of EUs assessed during 2017–2022 integrated transmission assessment surveys in Haiti

EU	Department	District(s)	Population (2023 estimate)	Baseline CFA Prevalence, 2001 (%)	Years of LF MDA	Number of LF MDA Rounds
1	Center	Saut d’Eau	43,616	44.0	2002–2012	8
2	Center	Hinche, Maissade, Thomonde, Cerca Cavajal, Mirebalais, Boucan Carré, Lascahobas, Belladère, Savanette, Cerca-la-Source, Thomassique	789,463	1.1	2011–2013	3
3	West	Petion-Ville	420,688	4.0	2012–2016	5
4	West	Pointe-à-Raquette	27,371	1.0	2009–2018	8
5	West	Anse-à-Galets	69,839	7.0	2009–2018	8
6	South	Camp-Perrin	50,285	7.0	2002–2012	11
7	South	Baradères, Cayes, Torbeck, Chantal, Maniche, Ile à Vache, Port Salut, Saint Jean du Sud, Arniquet, Aquin, Saint Louis du Sud, Cavaillon, Côteaux, Port à Piment, Roche à Bateau, Chardonnières, Les Anglais, Tiburon	860,925	1.2	2011–2015	5
8	Grande Anse	Jérémie, Abricots, Bonbon, Moron, Chambellan, Anse d’Hainault, Dame Marie, Les Irois, Corail, Roseaux, Beaumont, Pestel	522,798	0.5	2011–2015	5
9	North	Limbe	95,229	19.0	2004–2014 2017–2018	10
10	North	Grande-Riviere du Nord, Saint-Raphael, La Victoire, Pignon,* Bahon, Ranquite	223.138	2.9	2008–2015	7
11	North West	La Tortue Island	43,391	6.0	2003–2004	2

CFA = circulating filarial antigen; EU = evaluation unit; LF = lymphatic filariasis; MDA = mass drug administration.

* Pignon district in EU10 received only six rounds of MDA from 2008 to 2013.

Transmission assessment surveys were conducted according to WHO procedures.^7^ Briefly, children 6–7 years old were tested for LF in cross-sectional community-based (12 EUs) or school-based (three EUs) cluster surveys depending on the school attendance for 6- and 7-year-old children at the time of the survey. Community-based TASs are recommended when the net primary school enrollment ratio is <75%.^7^ The target sample size for each EU was calculated using the Survey Sample Builder (SSB).^33^ Sample sizes ranged from 506 to 1,556 with critical cutoff values of 6–18 CFA-positive children. The TAS sample sizes and critical cutoff values were powered so that an EU had at least a 75% chance of passing if the true antigen prevalence was 1.0% and no more than about a 5% chance of passing (incorrectly) if the true antigen prevalence was ≥2.0%, the level below which *Culex-*transmitted–*W. bancrofti* is believed to be unsustainable.^7^

For school-based TASs, 40 schools (clusters) along with a list of 10 backup schools were randomly selected for each EU using the SSB from a comprehensive list of primary schools (both public and private) provided by the Haitian Ministry of Education and the community. At each selected school, teachers identified and invited all children 6–7 years old to participate. For the community-based TASs, 30–87 targeted clusters along with a list of 10–20 backups were randomly selected for each EU using the SSB from a list of localities provided by the Haitian Institute of Statistics and Informatics (Institut Haitien de Statistique et d’Informatique). Survey teams visited selected households using a sampling interval in each selected community. If teams visited a household and there was nobody home, they moved to an adjacent house and did not plan to return to the empty one. At each participating household, all present children 6–7 years old were invited to participate.

### Blood testing.

Finger prick blood samples were collected by trained technicians using aseptic procedures from all assenting children and were tested on-site for the presence of CFA by Filariasis Test Strip (FTS; Abbott, Inc., Scarborough, ME) according to the manufacturer’s instructions. Results were read at 10 minutes, recorded on paper forms, and communicated confidentially to each child as well as to the school headmaster (school-based TASs) or parents (community-based TASs). All invalid FTS results and positive tests were repeated for confirmation. A third test was performed in cases of a discordant or inconclusive result and was considered the final result. All antigen-positive children were offered DEC-albendazole according to national policy. Follow-up testing for nocturnal MF was not performed. All assenting children also were tested for *Plasmodium* spp. infection by CareStart^™^ Malaria HRP-2/pLDH (Pf/pan) Combo Test (Access Bio Inc., Somerset, NJ) according to the manufacturer’s instructions. The only exceptions were the TAS-2 s in Pointe-à-Raquette and Anse-à-Galets in 2022, where malaria testing was not conducted because of unavailability of rapid diagnostic tests (RDTs). Results were read in 20 minutes, recorded, and communicated to each participant as well as to their teacher, parents, and district health officer for follow-up and treatment according to national malaria policy.

### Data collection and analysis.

All surveys were programmed into NEMO, The Carter Center’s open-source data collection software. Data were collected on paper and also submitted electronically using Android phones. At each school or household, survey data including global positioning system coordinates and demographics were collected using Android phones. Owing to the duration of the tests and potential need for repeated tests, FTS and RDT results were first recorded on paper and then entered electronically after all results were recorded for a single child. Unique identifiers were assigned to each child and used for data entry to protect privacy. Data were analyzed using either Microsoft Excel (Redmond, WA) or Stata (StataCorp, College Station, TX).^34^

### Ethical approval and consent procedures.

Transmission assessment surveys were conducted as nonresearch public health evaluations by the Haitian MSPP. District health officers identified community leaders to inform selected communities about the TAS activities and to accompany survey teams. Written informed consent was obtained from parents or school headmasters at each selected household and school, respectively. Verbal assent was also obtained from each child before finger prick blood sampling.

## RESULTS

### Lymphatic filariasis.

A total of *N* = 18,947 children with valid LF test results participated in 15 TASs conducted in 11 EUs. For TAS-1 s, a total of *n* = 5,239 children were tested across four EUs, each representing a single district (Table 2). The actual sample size (range: 892–1,560) exceeded the target sample size in each EU. Eight children (0.15%) tested positive for CFA. The number of CFA-positive individuals in each EU (range: 0–4) was less than the cutoff (range: 11–18), meaning that each EU passed TAS-1. Geographically, the positive samples were widely distributed in all four EUs, and no cluster contained more than one positive sample.

**Table 2:** Summary of TAS-1 results for lymphatic filariasis by filariasis test strip and *Plasmodium falciparum* malaria by rapid diagnostic test by evaluation unit in four districts, Haiti

Year	EU	Department	District	LF					Malaria		Survey Type
				Target Sample Size	Children Tested, FTS, No.	Positive FTS, No. (%)	Critical Cutoff	TAS Result (Pass/Fail)	Children Tested, RDT, No.	PF-Positive, RDT, No. (%)	
2017	3	West (Metropolitan Area)	Petion-Ville	1,556	1,560	0 (0.0)	18	Pass	1,560	0 (0.0)	Community
2019	4	West	Pointe-à-Raquette	891	892	2 (0.2)	11	Pass	892	1 (0.1)	Community
2019	5	West	Anse-a-Galets	1,380	1,403	4 (0.3)	16	Pass	1,403	2 (0.1)	Community
2019*	9	North	Limbe	1,380	1,384	2 (0.1)	16	Pass	1,384	0 (0.0)	Community
Subtotal	4	2	4	5,207	5,239	8 (0.2)	–	–	5,239	3 (0.1)	–

EU = evaluation unit; FTS = filariasis test strip; LF = lymphatic filariasis; PF = *Plasmodium falciparum*; RDT = rapid diagnostic test; TAS = transmission assessment survey.

* Repeat TAS-1.

For PTS TAS-2 s, a total of *n* = 11,866 children were tested across nine EUs covering 52 communes (Table 3). The actual sample size (range: 653–1,658) exceeded the target sample size in each EU. Twenty children (0.17%) tested positive for CFA. The number of CFA-positive individuals in each EU (range: 0–7) was less than the cutoff (range: 6–18), meaning that each EU passed TAS-2. The greatest number of positive children (seven) was found in Grande Anse, where the cutoff was 18. No cluster contained more than one positive sample.

**Table 3:** Summary of TAS-2 results for lymphatic filariasis by filariasis test strip and *Plasmodium falciparum* malaria by rapid diagnostic test by evaluation unit in 52 districts, Haiti

Year	EU	Department	Districts	LF					Malaria		Survey type
				Target Sample Size	Children Tested, FTS, No.	Positive, FTS, No. (%)	Critical Cutoff	TAS Result (Pass/Fail)	Children Tested, RDT, No.	PF Positive, RDT, No. (%)	
2018	1	Center	Saut d’Eau	909	918	4 (0.4)	11	Pass	918	6 (0.7)	Community
2019	2	Center	Belladeres, Boucan Carre, Cerca Cavajal, Cerca-La-Source, Hinche, Lascahobas, Maissade, Mirebalais, Savannette, Thomassique, Thomonde	1,556	1,571	0 (0.0)	18	Pass	1,573	5 (0.3)	Community
2019	3	West (Metropolitan Area)	Petion-Ville	1,556	1,555	2 (0.1)	18	Pass	1,555	0 (0.0)	Community
2019	6	South	Camp Perrin	506	653	2 (0.3)	6	Pass	653	0 (0.0)	School
2019	7	South	Cayes, Aquin, Torbeck, Saint Louis du Sud, Cavaillon, Barraderes, Port Salut, Chantal, Les Anglais, Saint Jean du Sud, Chardonnieres, Tiburon, Coteaux, Port A Piment, Roche A Bateau, Iles A Vache, Arniquet, Maniche	1,556	1,579	1 (0.1)	18	Pass	1579	1 (0.1)	School
2019	8	Grande Anse	Jeremie, Pestel, Dame Marie, Abricot, Anse D’Hainault, Roseaux, Corail, Moron, Chambelan, Les Irois, Beaumont, Bonbon	1,552	1,642	7 (0.4)	18	Pass	1,644	4 (0.2)	School
2019	10	North	Grande-Rivière du Nord, Bahon, Pignon, Saint Raphael, Ranquitte, La Victoire	1,532	1,658	2 (0.1)	18	Pass	1,660	3 (0.2)	Community
2022	4	West	Pointe-a-Raquette	780	854	1 (0.1)	11	Pass	N/A	–	Community
2022	5	West	Anse-à-Galets	1,368	1,436	1 (0.1)	16	Pass	N/A	–	Community
Subtotal	9	5	52	11,325	11,866	20 (0.2)	–	–	9,582	19 (0.2)	–

EU = evaluation unit; FTS = filariasis test strip; LF = lymphatic filariasis; PF = *Plasmodium falciparum*; RDT = rapid diagnostic test; TAS = transmission assessment survey.

For PTS TAS-3 s, a total of *n* = 1,842 children were tested across two EUs, each representing one commune (Table 4). The actual sample size (range: 918–924) exceeded the target sample size in each EU. Eight children (0.43%) tested positive for CFA. The number of CFA-positive individuals in each EU (range: 3–5) was less than the cutoff, meaning that each EU passed TAS-3, and no cluster contained more than one positive sample.

**Table 4:** Summary of TAS-3 results for lymphatic filariasis by filariasis test strip and *Plasmodium falciparum* malaria by rapid diagnostic test, by evaluation unit in two districts, Haiti

Year	EU	Department	Districts	LF					Malaria		Survey Type
				Target Sample Size	Children Tested, FTS, No.	Positive FTS, No. (%)	Critical Cutoff	TAS Result (Pass/Fail)	Children Tested, RDT, No.	PF Positive, RDT, No. (%)	
2020	1	Center	Saut d’Eau	911	924	5 (0.5)	11	Pass	924	5 (0.5)	Community
2020	11	North West	La Tortue Island	911	918	3 (0.3)	11	Pass	918	4 (0.4)	Community
Subtotal	2	2	2	1,822	1,842	8 (0.4)	–	–	1,842	9 (0.5)	–

EU = evaluation unit; FTS = filariasis test strip; LF = lymphatic filariasis; PF = *Plasmodium falciparum*; RDT = rapid diagnostic test; TAS = transmission assessment survey.

### Malaria.

A total of *n* = 16,663 children were tested for malaria by RDT during LF TASs, of whom 31 (0.19%) tested positive: three (0.06%) of *n* = 5,239 children in TAS-1 (Table 2), 19 (0.20%) of *n* = 9,582 children in TAS-2 (Table 3), and nine (0.49%) of *n* = 1,842 children in TAS-3 (Table 4). Geographically, children testing RDT positive were widely distributed. Only one cluster (EU1 in TAS-3) contained two RDT-positive samples. No other cluster contained more than one positive sample.

## DISCUSSION

This study determined that WHO criteria to stop MDA for LF were achieved in each of four communes encompassing 571,358 people for whom TAS-1 was conducted, meaning that cumulatively 122 (87%) of 140 communes in Haiti have stopped MDA and have progressed to PTS. Notably, this includes Limbe, the first commune in the country to pass a repeated TAS-1 after remedial rounds of MDA with extended social mobilization with radio spots, megaphones and a sound truck and mop-up. Limbe initially passed TAS-1 in 2015, after 10 years of MDA exceeding 65% reported coverage, but failed TAS-2 in 2017. After failing, two additional rounds of MDA with reported coverage of 104% in 2017 and 2018 were provided before Limbe successfully passed a pre-TAS then a TAS-1 in 2019 as reported here. This indicates that several limited rounds of MDA may be sufficient to (re-)suppress transmission in areas where LF recrudescence or importation may have occurred. Several other communes in Haiti (Dondon, Cap-Haïtien, Limonade, Plaine du Nord) also have required remedial MDA after previously stopping, but repeated TAS-1 s have not yet been completed in these areas. These data will be important to further determine effective and efficient remedial MDA approaches.

The study also found that all study areas passed TAS-2 and TAS-3 PTS surveys. These results demonstrate the effectiveness of the strategies used to suppress transmission beneath putative transmission breakpoints, although the risk of recrudescence or importation remains, especially in areas close to those where transmission is ongoing. Studies from American Samoa,^35^^,^^36^ Sri Lanka,^37^^,^^38^ and Zanzibar,^39^^,^^40^ along with unpublished data from Haiti, revealed sustained or even increased *W. bancrofti* transmission after successfully passing one or more TASs. One potential factor is that TASs only recommend sampling of children 6–7 years old, who likely have different exposure risks than adults and older children. It is possible that the TAS sampling strategy may not be sensitive to detect ongoing transmission in communities. Population mobility also presents a risk in Haiti, as people move from one commune to another for reasons that include education, business, and displacement caused by natural disaster or insecurity. These factors highlight the importance of robust surveillance during PTS, especially in areas where these contextual factors exist or where signals indicate persistent transmission. Recent operational research in Haiti described several options for conducting follow-up investigations of CFA-positive cases identified during a TAS.^41^

This study documents the first set of communes in Haiti to complete and pass TAS-3. However, sustained antigen positivity in both EUs is of significant concern. Of key importance is discerning whether CFA positivity in TAS-3 represents active transmission in an EU. Despite a similar number of CFA-positive individuals in both La Tortue Island (three) and Saut d’Eau (five), further examination of the available data suggests different conclusions. The three CFA-positive individuals in La Tortue Island lived in nearby but unique survey clusters in the same section communale (i.e., the smallest subdistrict administrative unit), potentially signaling local transmission. However, external exposure is more likely as all three children reported significant travel to Port-de-Paix, an endemic area on the mainland, and no CFA-positive children were found in TAS-2 in La Tortue Island (unpublished data; MSPP). In contrast, results from TAS-2 (four CFA-positive individuals) and TAS-3 (five CFA-positive individuals, all of whom lived in different clusters across four section communales) in Saut d’Eau are suggestive of sustained low-level transmission. Alternatively, CFA positivity could simply reflect infection with a single adult parasite that does not pose a transmission threat as the parasite population dwindles within communities. Confirmatory MF testing would reveal current infection status and guide programmatic responses to TAS-3 signals. Unfortunately, MF testing was not a part of these TAS protocols. Although the WHO recommends continued surveillance after passing TAS-3, there are currently no specific guidelines for required approaches.^42^ The MSPP is considering options that include additional TASs or TAS-like surveys, follow-up of antigen-positive clusters identified in TASs, continued sentinel and spot-check site monitoring, integration of LF antigen or serological testing within cross-sectional surveys for other diseases, and xenomonitoring. Unfortunately, these activities have not been possible because of insecurity in Haiti. From 2017 to 2023 the national malaria control program distributed nationwide approximately 1.7 million long-lasting insecticidal nets, which provide protection against nighttime biting mosquitoes that transmit malaria and LF.

Results from integrated malaria testing confirmed the low prevalence of *P. falciparum* malaria across Haiti. The overall RDT-based *P. falciparum* infection prevalence of 0.19% among young children in TASs is consistent with PCR-based prevalence estimates of <1% from large-scale malaria surveys in Haiti,^26^^–^^29^ as well as integrated LF-malaria TASs.^32^ However, transmission is heterogeneous in both space and time, and large-scale surveys may miss hotspots of transmission in low-prevalence settings such as Haiti.^43^

Saut d’Eau district in Center department was repeatedly found to have the highest malaria prevalence across surveyed areas (0.65% in 2018 and 0.54% in 2020), suggesting a focal area of transmission. It was surprising that estimates for Saut d’Eau exceeded those of Grande Anse (0.24% in 2019), an area of known high transmission.^26^^,^^28^^,^^31^ One possible explanation is interannual variation: more than twice the number of malaria cases were reported nationwide in 2020 (22,987) compared with 2019 (10,687 cases).^44^ This may also explain the higher prevalence observed in La Tortue Island in 2020 (0.44%). Seasonal variations may also account for some differences depending on the timing of integrated TASs, which were generally conducted between April and September each year. Integration of malaria testing with other public health assessments increases the cost efficiency of surveillance for malaria elimination, with a previous study in Haiti determining that the addition of malaria RDT added only 15% extra costs to a standard LF TAS, whereas additional gains were realized in cross-training of health workers and reduced disruption for targeted communities.^32^ After similar approaches to integrated LF-malaria surveys in the Dominican Republic,^45^ this study appears to be the first to use multi-species malaria RDTs in Haiti, an important aspect given the persistent threat for *Plasmodium vivax* importation from other countries in the region and the recent detection of *Plasmodium malariae* in Haiti.^46^

This study faced several limitations: Insecurity in Haiti posed a persistent threat to study teams and the local population. Additional TASs were planned but could not be conducted safely. Population movements due to instability may have biased the representativeness of samples in each EU by omitting previous inhabitants and/or by including recently arrived inhabitants. The EUs were formed from geographically and epidemiologically similar IUs with a total EU population of no more than 2 million residents (the largest EU represented a population of 860,925), in compliance with current WHO guidelines.^7^ However, the aggregation of multiple IUs into a single EU may have increased the risk of missing small foci of transmission. For example, five of seven CFA-positive individuals in the TAS-2 in EU8 (Grande Anse) were located in one commune. If resources permit, evaluation of individual IUs for subsequent TASs would provide higher resolution monitoring data and a greater likelihood of identifying potential microfoci of transmission.^47^ In addition, school-based sampling may not be entirely representative of the target IU, as children may be enrolled in a school that is located in a different IU from where they reside. As mentioned above, follow-up testing for MF was not conducted to confirm whether CFA-positive participants harbored active infections. Malaria prevalence estimates likely underestimated the true infection prevalence, as testing was conducted only by RDT, which is less sensitive than PCR testing.

In conclusion, these results provide evidence of significant progress in stopping MDA for LF in Haiti and maintaining levels below the threat of a public health problem. At the same time, they reveal the challenge and importance of reaching total interruption of transmission so that interventions can be safely and permanently halted. Similarly, these surveys show the feasibility of integrating malaria testing within LF assessments, which provides a cost-effective surveillance strategy for monitoring progress toward elimination of both diseases in resource-limited environments such as Haiti.
